# Sleep quality, social rhythms, and depression among people living with HIV: a path analysis based on social zeitgeber theory

**DOI:** 10.3389/fpsyt.2023.1102946

**Published:** 2023-05-04

**Authors:** Jingjing Meng, Xueling Xiao, Wenru Wang, Ying Jiang, Yanfei Jin, Honghong Wang

**Affiliations:** ^1^Xiangya School of Nursing, Central South University, Changsha, Hunan, China; ^2^Alice Lee Centre for Nursing Studies, Yong Loo Lin School of Medicine, National University of Singapore, Singapore, Singapore

**Keywords:** sleep quality, social rhythms, depression, HIV/AIDS, path analysis

## Abstract

**Background:**

People living with HIV frequently report sleep disturbances. The social zeitgeber theory, which proposes that stressful life events can interfere with sleep and even depression by destabilizing daily routines, provides new insights into identifying predictors of sleep disturbances and improving sleep in people living with HIV.

**Objective:**

To explain the pathways affecting sleep quality in people living with HIV based on social zeitgeber theory.

**Methods:**

A cross-sectional study was conducted to assess sleep quality, social rhythms, depression, social support, and coping styles from December 2020 to February 2021. The hypothetical model was tested and respecified by performing path analysis and a bias-corrected bootstrapping method using IBM AMOS 24 software. The report of this study followed the STROBE checklist.

**Results:**

A total of 737 people living with HIV participated in the study. The final model presented a good fit (goodness of fit = 0.999, adjusted goodness of fit index = 0.984, normed fit index = 0.996, comparative fit index = 0.998, Tucker–Lewis index = 0.988, root mean square error of approximation = 0.030, chi-squared/degree of freedom = 1.646), explaining 32.3% of the variance in sleep quality among people living with HIV. Lower social rhythm stability was directly associated with poorer sleep quality, and depression mediated the relationship between social rhythms and sleep quality. Social support and coping styles affected sleep quality through social rhythms and depression.

**Limitation:**

The cross-sectional study design precludes making assumptions about causality among factors.

**Conclusion:**

This study validates and extends the applicability of the social zeitgeber theory in the HIV context. Social rhythms have direct and indirect effects on sleep. Social rhythms, sleep, and depression is not simply linked in a cascading sequence but is theoretically linked in a complex way. More studies are needed to explore the predictors of social rhythms, and interventions for stabilizing social rhythms have the potential to alleviate sleep disturbances and depression in people living with HIV.

## Introduction

People living with HIV (PLWH) frequently report sleep disturbances. A meta-analysis showed that the prevalence of self-reported sleep disturbances in PLWH was 58% (1). The sleep disturbances reported by PLWH mainly included reduced total sleep time, difficulty falling asleep, difficulty maintaining sleep, sleep reinitiation disorder, daytime dysfunction, excessive dreaming, and irritability (2–4). Sleep disturbances can contribute to a series of adverse consequences for PLWH, such as greater depressive symptoms (5), poorer health-related quality of life (6), increased risk of incident cardiovascular disease (7), lower HIV medication adherence, and self-reported increased HIV symptom severity (8).

Several antecedents of sleep disturbances in the HIV context were identified, including a long duration of HIV infection (9), disease progress (10), HIV treatment regimen containing efavirenz (11), and multiple psychosocial factors, such as being single (12), being unemployed (13), unsatisfactory lifestyles (14), depression (9), internalized HIV stigma (15), and poor social support (16). However, disease-related antecedents are difficult to change. Acquired immune deficiency syndrome (AIDS) is currently not curable, and PLWH need to take antiretroviral therapy medications regularly and even for life (17). Sleep disturbances remain severe even in groups of PLWH with successful virological suppression (18). Several studies have attempted to improve the sleep of PLWH by correcting sleep-related inappropriate behaviors (19–21), but the efficacy of interventions is controversial, and none of these interventions has been developed in the HIV-specific context. Therefore, it is necessary to further explore modifiable factors that affect the sleep of PLWH, clarify the relationships between these factors, and the pathways through which they affect sleep. This is conducive to understanding the mechanism of sleep disturbances in PLWH, developing targeted intervention strategies, and interventions aimed at modifiable psychosocial attributes that may alleviate sleep disturbances in PLWH.

## Theoretical background

The human biological clock needs to remain synchronized to seasonal variations in the timing of sunrise and sunset. Environmental cues providing this synchronization to the biological clock are known as zeitgebers, and light is the most potent zeitgeber (22). However, “social zeitgebers,” such as awakening in the morning, exercising, mealtimes, social demands, and other activities within typical daily routines, also impact the circadian system (23). Social rhythms refer to the regularity of these social and lifestyle activities that act as social zeitgebers (24). Studies have shown that subjects with higher lifestyle regularity and more daily social contacts reported fewer sleep problems (25, 26) and that interventions focused on stabilizing social rhythms can significantly improve sleep quality in patients with mental disorders (27, 28). The social zeitgeber theory (29) proposes one possible explanation for the relationship between sleep and social rhythms. The theory posits that critical life events can produce changes in social zeitgebers that destabilize social rhythms, which in turn leads to instability in specific biological rhythms, especially sleep, and in vulnerable individuals, can even further lead to severe depressive episodes.

Evidence for the social zeitgeber hypothesis is incomplete, and no study to date has tested this theory in PLWH. The existing research on social rhythms mainly focuses on the link with bipolar disorder (30), and only a few studies investigate a certain aspect of social zeitgebers in PLWH, such as physical activity and exercise (31–33). Researchers found PLWH had poor sleep regularity (31) and lower amounts of physical activity compared to people living without HIV (32). Nearly 66.0% of PLWH did not exercise regularly, and they were more likely to suffer sleep disturbances than PLWH who reported regular exercise (33). PLWH suffer from multiple psychosocial pressures, such as stigma, social rejection, health concerns, and unemployment (34, 35), which make them more likely to deviate from their original life trajectories and increase the risk of unstable social rhythms. However, the level of social rhythms in PLWH and how social rhythms are linked to sleep disturbances in the HIV context remains unclear.

The conceptual framework of the present study was organized by aggregating factors in the social zeitgeber theory as well as existing evidence. The social zeitgeber theory posits that the instability of social rhythms may disrupt sleep and even cause depression (29). It should be noted that the theory was proposed to explain the pathogenesis of depression (29) and that existing studies testing the theory and examining the cascading sequence relationship between social rhythms, sleep, and depression have mainly focused on bipolar disorder or depression (36–38), while the relationship between these three variables in PLWH remains unclear. The global prevalence of depression among PLWH is as high as 31.0% (39). Studies have found a complex bidirectional relationship between sleep disturbances and depression (40, 41). In PLWH, sleep disturbance was a significant predictor of depression (5, 42), while depression was also a risk factor for sleep disturbances (43). Therefore, we hypothesized a bidirectional relationship between depression and sleep in PLWH. Although the primary path of social zeitgeber theory hypothesizes only the relationship between social rhythms, sleep, and depression, the theory also suggests examining the effects of factors such as social support and coping styles as potential intervening variables on the stability of social rhythms within the model (29). Therefore, we hypothesized that social support and coping styles directly affect social rhythms and indirectly affect sleep through social rhythms.

**Figure 1:**
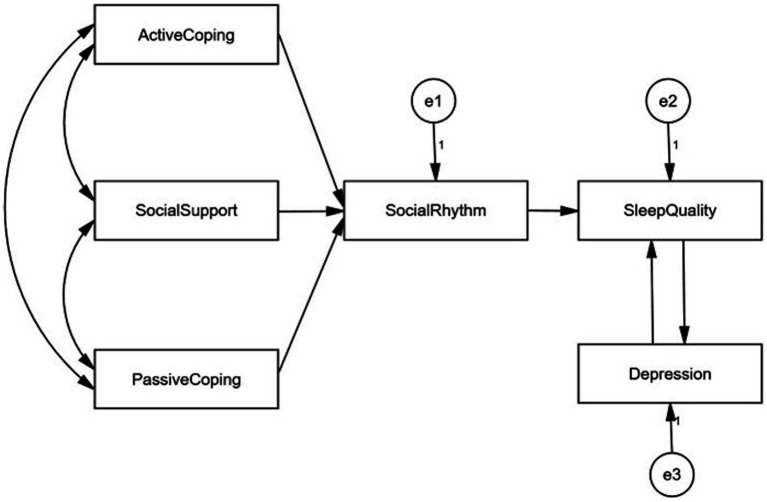
The hypothetical model base on social zeitgeber theory.

The current study attempted to investigate a theoretically plausible link by identifying predictor variables of sleep in PLWH, including social rhythms, depression, social support, and coping styles. Based on the social zeitgeber theory and existing evidence, we propose the following four hypotheses (1): Social rhythms directly predict sleep quality (2), social support and coping styles (active coping, passive coping) directly predict social rhythms (3), social support and coping styles (active coping, passive coping) indirectly predict sleep quality through social rhythms, and (4) there was a bidirectional predictive association between sleep and depression. The hypothetical model is presented in Figure 1. This study will provide evidence to shed light on the sleep of PLWH from the perspective of social rhythms by including other psychosocial factors (social support, active coping, passive coping, and depression) and further imply future intervention development.

## Materials and methods

### Design

This is a cross-sectional study to investigate sleep quality and its influencing factors in PLWH, which is part of a sleep promotion project called “AI Youmian” (Love Better Sleep) in Changsha, Hunan, China. The study was conducted and reported followed the Strengthening the Reporting of Observational Studies in Epidemiology (STROBE) checklist (44).

### Sample

Participants were recruited from the HIV clinics of the First Hospital of Changsha City and the Center for Disease Control and Prevention of Yuelu District, Hunan Province, China, from December 2020 to February 2021. The First Hospital of Changsha City is a designated third-class hospital for AIDS diagnosis and treatment, while the Center for Disease Control and Prevention is responsible for the counseling and management of PLWH.

The study population included those who were admitted to the HIV clinics for treatment or counseling during the data collection period and fulfilled the following eligibility criteria (1): had been diagnosed with HIV infection by HIV antibody testing or HIV nucleic acid testing (2), were age 18 years or older, and (3) were willing to join the study voluntarily. Individuals whom psychiatrists had assessed as having depression or bipolar disorder were excluded from the study.

### Sample size calculation

The sample size was estimated using the module of the confidence intervals (CIs) for one proportion of the PASS software (version 11). The prevalence of sleep disturbances in PLWH was 43.1%, attributable to a similar large-scale study in China conducted in 2017 (12). Assuming a 95% confidence level (*α* = 0.05, two-sided), a 0.08 CI width (two-sided), and a 15.0% non-response rate, the minimal sample size required was 719 participants.

### Measures

#### Sociodemographic variables

The demographic characteristics assessed included age, gender, living area, marital status, route of HIV infection, and being on antiretroviral therapy or not. Considering the factors within social zeitgeber theory, the variables tested in the present study included sleep quality, social rhythm, depression, social support, and coping styles.

#### Sleep quality

Sleep quality was assessed using the translated Chinese version of the Pittsburgh Sleep Quality Index (PSQI), which was translated and validated by Liu et al. (45) and was confirmed to have good reliability and validity in Chinese PLWH (46). The original scale was an English version designed by Buysse et al. (47) to measure sleep quality and disturbances over the previous month and can be used to identify patients with sleep disturbances in both general clinical and psychiatric settings. The scale consists of 18 items divided into seven dimensions, including subjective sleep quality, sleep duration, sleep efficiency, sleep latency, sleep disturbances, daytime dysfunction, and use of sleep medication. The total score of the scale is the sum of the scores of each dimension. Each dimension is scored from 0 to 3. The total PSQI score ranges from 0 to 21, with higher scores indicating poorer sleep quality. A total PSQI score of >5 indicates sleep disturbances (47), while in the Chinese population, a total PSQI score > 7 indicates sleep disturbances (45). Validation of the measurement model resulted in a fit of the chi-squared/degree of freedom (*χ*^2^/df) = 1.995, the goodness of fit index (GFI) = 0.991, comparative fit index (CFI) = 0.989, Tucker–Lewis index (TLI) = 0.980, and root mean square error of approximation (RMSEA) = 0.037. In the current sample, this scale’s Cronbach’s alpha was 0.74, and the composite reliability (CR) was 0.73.

#### Social rhythm

The regularity of daily activities was assessed using the Brief Social Rhythm Scale (BSRS) designed by Margraf and colleagues (48). The original language of the BSRS is English, and the Chinese version was translated by Wu (49). The scale consists of 10 items to assess the regularity of daily routines during the week, including activity rhythm (waking, bedtime, and diet) and social interaction rhythm (work/study and leisure time). Participants were asked to rate the regularity of the above activities, such as the regularity of “Meeting other people in my free time Mondays through Fridays.” The regularity of each activity is rated on a six-point Likert scale and scored from 1 (very regular) to 6 (very irregular). The total score of the scale is obtained by adding up the scores of each item. The total BSRS score ranges from 10 to 60, with higher total scores reflecting greater irregularity in daily activities. The overall verification results of the measurement model were as follows: χ^2^/df = 5.025, GFI = 0.989, CFI = 0.995, TLI = 0.972, and RMSEA = 0.074. The BSRS showed good reliability in a previous study (50), Cronbach’s alpha was 0.90 and 0.81, and the CR was 0.92 and 0.83 in our sample.

#### Depression

Depression severity was investigated using the Self-Rating Depression Scale (SDS) compiled by Zung (51), which has been widely used and validated in China (52, 53). The original language of the scale is English, and the Chinese version was translated by Wang et al. (54). The scale included 20 items, and each item was scored on a four-point Likert scale to examine the frequency of the occurrence of the assessed symptoms over the previous week (1 = A little of the time, 2 = Some of the time, 3 = Good part of the time, 4 = Most of the time). Of the 20 items, 10 items assess negative symptoms, such as “I feel down-hearted and blue,” and 10 items assess positive symptoms, such as “eat as much as I used to.” The total raw score is the sum of the negative scores of 10 items and the reversed positive scores of 10 items. The total raw score ranged from 20 to 80. The standardized score was the original total score multiplied by 1.25; a score of less than 50 is considered no depression, 50–59 minimal to mild depression, 60–69 moderate-to-marked depression, and 70 or more severe depression. Validation of the measurement model resulted in a fit of *χ*^2^/df = 3.020, GFI = 0.948, CFI = 0.931, TLI = 0.902, and RMSEA = 0.052. The Cronbach’s alpha coefficient of this scale in this study was 0.82, and the CR of this scale was 0.87.

#### Social support

Social support was assessed using the Perceived Social Support Scale (PSSS). The 12-item PSSS was an English-language scale developed by Zimet et al. (55) and assesses perceived support from three groups–family, friends, and significant others–such as “I have friends with whom I can share my joys and sorrows.” The total score of the scale is obtained by adding up the scores of each item. The scale uses a seven-point Likert scale, with total scores ranging from 12 to 84 and higher scores indicating higher levels of perceived social support. The Chinese version of the PSSS this study used was translated by Huang et al. (56) and showed good internal reliability. Validation of the measurement model resulted in a fit of *χ*^2^/df = 2.880, GFI = 0.976, CFI = 0.992, TLI = 0.986, and RMSEA = 0.051. In this study, Cronbach’s alpha value was 0.92, 0.95, and 0.92, and the CR was 0.92, 0.94, and 0.92, respectively.

#### Coping style

The simplified coping style questionnaire developed by Xie (57) was used to evaluate coping styles, and the original language of the scale was Chinese. The 20-item scale includes two dimensions of active coping (12 items) and passive coping (8 items). Active coping styles were assessed through items such as “seeing the good side of things,” whereas passive coping styles were assessed through items such as “relying on others to solve problems.” Each item is scored on a four-point Likert scale, with higher scores indicating greater active/passive coping styles. The mean scores for the active and passive items were the final scores for active and passive coping, respectively. The scale has been widely used and validated in China (58, 59). Validation of the measurement model resulted in a fit of *χ*^2^/df = 3.590, GFI = 0.933, CFI = 0.922, TLI = 0.901, RMSEA = 0.059, and Cronbach’s alpha values for active and passive coping were 0.88 and 0.73, respectively, and the CR was 0.87 and 0.73 in this study.

### Ethical considerations

For ethical consideration, the study was conducted following the tenets of the Declaration of Helsinki and was approved by the Institutional Review Board of Central South University (approval number: E2020182). The competent authorities of the First Hospital of Changsha City and the Center for Disease Control and Prevention both approved the collection of data in HIV clinics.

### Data collection

Consecutive sampling was conducted in HIV clinics. Five investigators (a HIV specialist, a psychotherapist, two Masters of Nursing, and a PhD of Nursing) received training on questionnaire filling methods and quality control before recruiting participants, with psychotherapists having years of qualification and experience in questionnaire development. The investigators were responsible for clarifying the purpose, content, requirements, and potential benefits and risks of the survey to the study participants, informing them that it was an entirely voluntary study and that they had the right to withdraw at any time with no impact on their receipt of medical services. Participants were also informed that the data were anonymous and presented in a total sample format to help alleviate their reluctance and concerns about privacy breaches. Informed consent was obtained from all the participants. For those with low education levels or who could not fill out the questionnaire by themselves, an investigator explained the meaning of each item and assisted them in completing it. Data collection for each participant lasted 20–30 min, and each participant received a reward of 20 CNY (USD 2.94) after completing the questionnaire. If any missing items were found, the investigator would ask the participant to fill them in if they wished to.

### Data analysis

The data were entered via double entry into Epidata version 3.1. A descriptive statistical analysis of subject characteristics and study variables, correlations between variables, and the internal consistency of measurements were analyzed using IBM SPSS version 25.0. Harman’s single-factor test was conducted to assess common method bias using SPSS 25.0, and confirmatory factor analysis was conducted to test the validity and composite reliability of the scales using Amos 24.0. Model identification, model estimation, and model modification were performed using IBM SPSS Amos version 24.0.

The categorical variable was described by frequency and proportion, and the continuous variable was described by mean and standard deviation. Correlations between study variables were tested using the Pearson correlation coefficient. Path analysis was performed to test the hypothetical model. Multicollinearity was assessed using the Pearson correlation coefficient between variables. Skew (<3) and kurtosis (<8) were examined to confirm that those study variables were normally distributed (60). To estimate the goodness of fit of the model, GFI and adjusted goodness of fit index (AGFI), normed fit index (NFI), CFI, TLI, and RMSEA were used. Values of GFI, AGFI, NFI, CFI, and TLI greater than 0.90 and 0.95 were considered as model marginal fit and good fit (61), respectively, while RMSEA values less than 0.08 and 0.05 indicated model marginal fit and good fit (62), respectively. Since the chi-squared (*χ*^2^) value is highly dependent on the sample size, we used the chi-squared/degree of freedom (*χ*^2^/df) ratio as a measure of model fit, with a ratio of less than five indicating an acceptable fit. If any of these statistics indicated a poor model fit, the model would be re-specified according to the estimates and the modification index provided in the Amos output. As the hypothetical model is non-recursive, we used the automated model specification search function of AMOS to explore whether there is a feedback loop between sleep quality and depression. We set the connection between sleep quality and depression as optional arrows and compared the fit of the models based on the following criteria: The values of the zero-based Browne–Cudeck criterion (BCC0) ≤ 2 and the zero-based Bayesian information criterion (BIC0) ≤ 2 indicate that there is no evidence that the model is not optimal (63). Squared multiple correlations (SMC) were used for the explanatory power of the endogenous variable. To identify the indirect effects further, a bias-corrected bootstrapping method with 5,000 resamples and a 95% CI was used (60). When bootstrapping lower and upper bounds do not cross zero, a mediating effect exists (64). All analyzes set a statistically significant threshold with *p* < 0.05 and two-tailed.

## Results

A total of 1,097 PLWH who potentially met the eligibility criteria were approached, 32 PLWH younger than 18 years were excluded through screening, and 287 PLWH refused to participate; reasons for refusal included no time (*n* = 105), no interest in the study (*n* = 83), concern about disclosure of privacy (*n* = 52), and unwillingness to state the reasons for refusal (*n* = 47). A total of 778 PLWH participated in the survey; 41 questionnaires were eliminated due to incomplete filling in or errors in logical inspection, and 737 valid questionnaires were finally returned in this survey, with an effective response rate of 94.7%.

### Validity and reliability

The scales used in this study are all widely acknowledged and commonly used scales that have been shown to have good reliability and validity in previous research. In this study, the model fit of all scales was acceptable, with Cronbach’s alpha and CR higher than 0.7, indicating that the reliability and validity of the measurement tools used in this study were acceptable.

### The test of common method bias

The data in this study were collected anonymously, and some items (Self-Rating Depression Scale) were set with reverse scoring items to control common method bias. In addition, the Harman single-factor test method was used to conduct exploratory factor analysis on all items related to sleep quality, social rhythm, depression, social support, and coping strategies in PLWH. A total of 14 common factors with eigenvalues greater than one were extracted, and the maximum common factor explained 17.8% of the overall variance, which is lower than the critical standard of 40%. Based on this, it can be concluded that there is no serious common method bias in this study, and it meets the requirements for path analysis.

### Sample characteristics

The sample characteristics of the participants are shown in Table 1. The mean age of the participants was 38.74 (SD = 13.66), the vast majority were male (88.1%), the vast majority transmitted sexually (92.1%), and most were unmarried (52.6%) or had no partner (15.2%). The vast majority of participants were receiving antiretroviral therapy (97.0%).

**Table 1:** Characteristics of participants.

Characteristics	Mean (SD) OR N	Proportion (%)
Age	38.74 (13.66)	
Gender		
Male	649	88.1
Female	88	11.9
Living area		
Urban	603	81.8
Rural	134	18.2
Marital status		
Unmarried	388	52.6
Separated, divorced, or widowed	112	15.2
Married	237	32.2
Route of HIV infection		
Homosexual sex	430	58.3
Heterosexual sex	291	39.5
Blood	16	2.2
Antiretroviral therapy initiation		
Yes	715	97.0
No	22	3.0

### Description and bivariate correlation of main variables

According to the sleep disturbance criteria of the Chinese population (PSQI >7), 19.9% (*N* = 147) of participants were poor sleepers, while according to Buysse’s criteria (PSQI >5), 39.3% (*N* = 290) of participants were poor sleepers. Based on a cutoff score of 50, 38.5% (*N* = 284) of participants reported depressive symptoms, with 23.7% (*N* = 175), 13.2% (*N* = 97), and 1.6% (*N* = 12) reporting minimal to mild, moderate-to-marked, and severe depressive symptoms, respectively, and 13.4% (*N* = 99) of participants had the comorbidity of sleep disturbances (Chinese criteria) and depression.

The mean scores and correlations among the main variables are shown in Table 2. Sleep quality was significantly positively correlated with social rhythms and passive coping styles and significantly negatively correlated with social support and active coping styles. Except for passive coping, which was not correlated with social rhythm, depression, and social support, all other variables had bivariate significant correlations. The skewness and kurtosis of all variables do not exceed the absolute value of 1, indicating all variables followed the normal distribution. The correlation coefficients between all variables did not exceed 0.7, indicating there was no multicollinearity among the variables.

**Table 2:** Means, standard deviations, and correlation coefficients of the study variables.

Variable	M ± SD	Sleep quality	Social rhythm	Depression	Social support	Active coping	Passive coping	Skewness	Kurtosis
Sleep quality	5.0 ± 3.24	1						0.787	0.593
Social rhythm	24.4 ± 9.51	0.347^**^	1					0.639	0.414
Depression	46.5 ± 10.99	0.509^**^	0.217^**^	1				0.318	−0.501
Social support	54.1 ± 14.41	−0.200^**^	−0.169^**^	−0.331^**^	1			−0.408	0.246
Active coping	1.8 ± 0.59	−0.157^**^	−0.166^**^	−0.392^**^	0.485^**^	1		−0.265	−0.145
Passive coping	1.3 ± 0.54	0.105^**^	0.031	0.056	0.035	0.331^**^	1	0.377	0.588

M, Mean.

SD, Standard deviation.

** *p* < 0.01.

### Model fit

**Figure 2:**
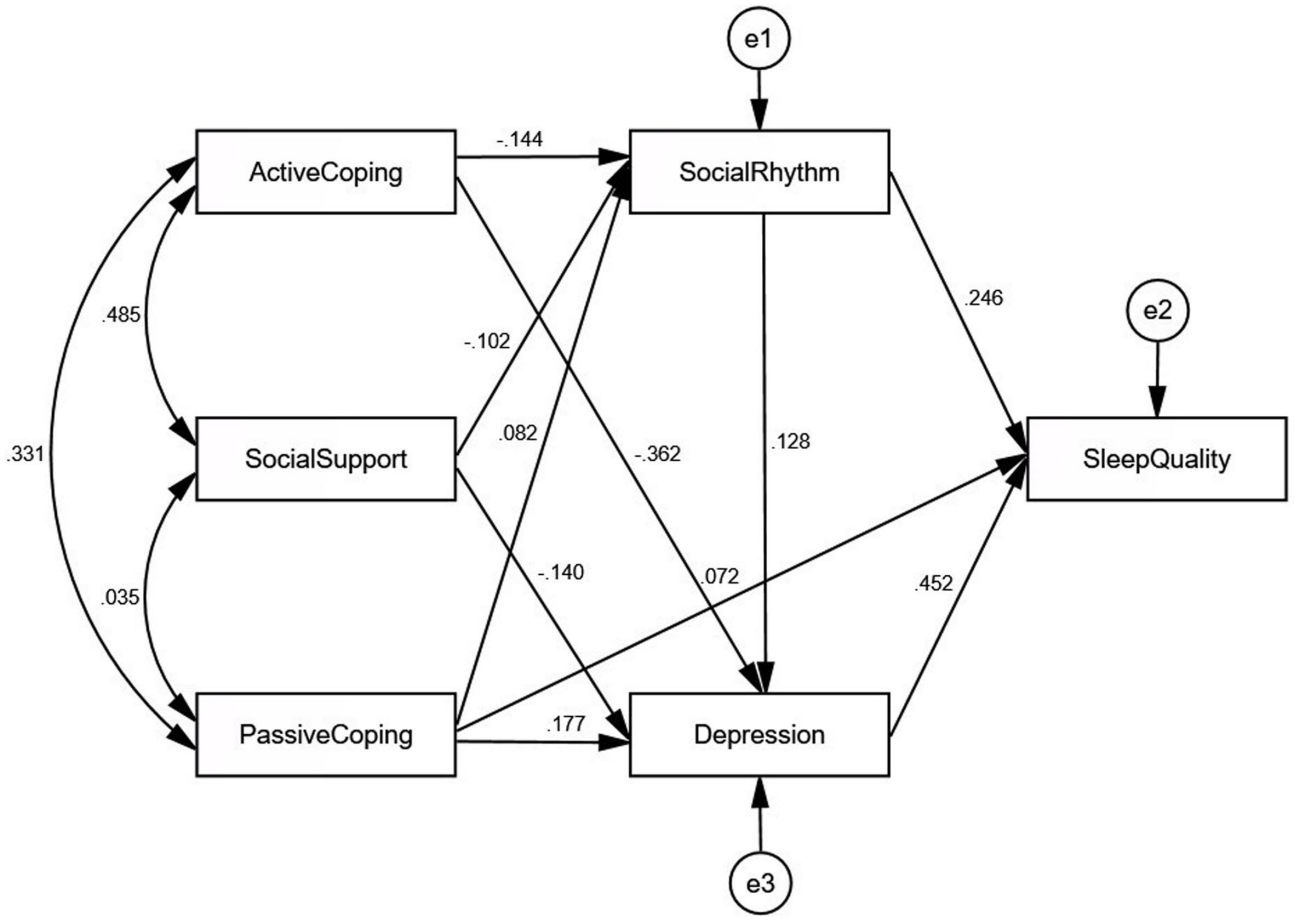
Modified model with standardized path estimates.

The initial model had a poor fit with the following goodness-of-fit indices: GFI = 0.938, AGFI = 0.782, NFI = 0.801, CFI = 0.805, TLI = 0.512, RMSEA = 0.188, and *χ*^2^/df = 26.954. The model was respecified by removing insignificant pathways and adding the suggested pathways with modification indices. We added paths between sleep quality and passive coping as well as paths between depression and social rhythms, social support, active coping, and passive coping, according to the descending order of the modification indices output. An automated model specification search was used to determine the relationship between sleep quality and depression. The path between sleep quality and depression was set as a two-way optional arrow, so four models were estimated for comparison, as follows: a two-way relationship, sleep quality affects depression, depression affects sleep quality, and no relationship. The model comparisons are shown in Table 3, and the results show that depression has a direct effect on sleep quality. The final model (Figure 2) showed good fit indices: GFI = 0.999, AGFI = 0.984, NFI = 0.996, CFI = 0.998, TLI = 0.988, RMSEA = 0.030, and *χ*^2^/df = 1.646. All path coefficients were significant in the final model, and the model explained 32.3, 22.6, and 4.4% of the variance in sleep quality, depression, and social rhythm, respectively.

**Table 3:** Comparison of model fitness.

Name	BCC 0	BIC 0	GFI	AGFI	NFI	CFI	TLI	RMSEA	χ^2^ /df
Depression→sleep	**0.000**	**0.000**	**0.999**	**0.984**	**0.996**	**0.998**	**0.988**	**0.030**	**1.646**
Non-recursive	0.966	5.549	0.999	0.979	0.997	0.998	0.977	0.041	2.238
Sleep→depression	20.984	20.984	0.989	0.888	0.970	0.972	0.790	0.123	12.138
No relationship	183.564	178.980	0.930	0.509	0.767	0.767	−0.166	0.290	62.958

BCC0, zero-based Browne–Cudeck criterion; BIC0, zero-based Bayesian information criterion; GFI, goodness of fit index; AGFI, adjusted goodness of fit index; NFI, normed fit index; CFI, comparative fit index; TLI, Tucker–Lewis index; RMSEA, root mean square error of approximation; χ^2^/df, chi-square /degree of freedom. The bold values indicate the fitting index for the best model.

### Path analysis

Table 4 shows unstandardized and standardized coefficients for direct paths between study variables, and Table 5 shows the bootstrapping analysis of the mediation effect significance test for the final model. Each path coefficient was significant in the model. The 95% confidence intervals of all the mediating pathways did not cross zero, and the standardized indirect effects are within the confidence intervals, indicating that all the mediation effects are significant.

**Table 4:** Path coefficients of study variables.

Model paths	Standardized estimates (*β*)	Unstandardized estimates	S.E.	C.R.	*P*
Active coping → Social rhythm	−0.144	−2.341	0.718	−3.263	0.001
Social support → Social rhythm	−0.102	−0.067	0.028	−2.436	0.015
Passive coping → Social rhythm	0.082	1.457	0.683	2.132	0.033
Passive coping → Depression	0.177	3.614	0.713	5.067	***
Social support → Depression	−0.140	−0.107	0.029	−3.706	***
Active coping → Depression	−0.362	−6.794	0.752	−9.035	***
Social rhythm → Depression	0.128	0.148	0.038	3.853	***
Depression → Sleep quality	0.452	0.133	0.009	14.529	***
Social rhythm → Sleep quality	0.246	0.084	0.011	7.934	***
Passive coping → Sleep quality	0.072	0.436	0.183	2.383	0.017

S.E., Standard error.

C.R., Critical ratio.

*** *p* < 0.001.

**Table 5:** Bootstrapped indirect effects on sleep quality and 95% confidence interval (CI) from the path analysis.

Model pathways	Standardized estimates	S.E.	Bias corrected 95% CI	
			Lower	Upper
Active coping → Social rhythm → Sleep quality	−0.036	0.012	−0.060	−0.015
Active coping → Depression → Sleep quality	−0.163	0.020	−0.205	−0.125
Active coping → Social rhythm → Depression → Sleep quality	−0.008	0.004	−0.018	−0.003
Social support → Social rhythm → Sleep quality	−0.025	0.013	−0.053	−0.002
Social support → Depression → Sleep quality	−0.063	0.020	−0.104	−0.026
Social support →Social rhythm → Depression → Sleep quality	−0.006	0.003	−0.014	−0.001
Passive coping → Social rhythm → Sleep quality	0.020	0.010	0.002	0.042
Passive coping → Depression → Sleep quality	0.080	0.017	0.048	0.114
Passive coping →Social rhythm → Depression → Sleep quality	0.005	0.003	0.001	0.012
Social rhythm → Depression → Sleep quality	0.058	0.016	0.026	0.090

S.E., Standard error.

CI, Confidence interval.

Social rhythm, depression, and passive coping style had significant direct effects on sleep quality. Social rhythms have a significant indirect effect on sleep quality through depression. Active coping styles, passive coping styles, and social support had significant indirect effects on sleep quality through social rhythms and depression.

## Discussion

To our knowledge, this is the first study to investigate the factors influencing sleep in PLWH based on social zeitgeber theory. Our findings revealed a high proportion of poor sleepers in PLWH, and the prevalence of sleep disturbances was much higher than that of the general population (7.6%) (65). Our results confirmed that social rhythms not only directly affect sleep in PLWH but indirectly affect sleep through depression. This study validates and extends the applicability of the social zeitgeber theory in the HIV context and provides new insights into the understanding of sleep disturbances in PLWH.

This study found that social rhythms predict sleep quality in PLWH, and hypothesis 1 was confirmed. Individuals with irregular lifestyles are less likely to be regularly exposed to light and follow stable bedtimes and wake times, and irregular daily routines might disrupt sleep by impairing robust circadian rhythms (25). In addition to daily activities such as sleeping and eating, social interaction is an important social zeitgeber as well (23). Due to HIV-related stigma and discrimination, PLWH deliberately reduce social contact or are forced to socially isolate (66, 67), and unsatisfactory or irregular social contact may contribute to poor sleep through circadian rhythm disruption.

We also found that social rhythms have an indirect effect on sleep quality through depression. Social rhythms have been linked to depression, and individuals diagnosed with depression show significant disturbances in their biological rhythm, particularly the sleep–wake cycle (36, 68). Previous studies have found that individuals with irregular lifestyles are more likely to experience emotional distress, such as depression, which can trigger or exacerbate sleep disturbance symptoms (69–71). Our study validates the cascading link between social rhythm, depression, and sleep quality in PLWH. Similarly, Brown et al. (72) found that the effects of social rhythm on sleep are influenced by the severity of depression, which suggests that depression may mediate the relationship between social rhythms’ stability and sleep. As the social rhythm factor is salient and modifiable, it could be targeted to potentially intervene with sleep disturbances and depression.

Our study identified the optimal pathway for the unidirectional effect of sleep quality on depression in PLWH using an automated model specification search function. We did not find a bidirectional relationship between sleep quality and depression, and hypothesis 4 was not confirmed. Previous studies have shown that circadian rest-activity rhythm is the strongest predictor of depression, which in turn affects sleep (73–75). Sleep disturbances are common complaints among PLWH with depression (76, 77) and can occur after depressive episodes and exacerbate depressive symptoms (78). Even after depressive symptoms subside, sleep disturbances can be residual symptoms that increase the risk of depression relapse (79). Social zeitgeber theory posits that depressive symptoms occur after changes in sleep (29). We speculate that the reason for this inconsistency depends on the context in which the theory is proposed. The social zeitgeber theory has been proposed to explain the pathogenesis of major depression (29), and existing studies have mainly tested this theory in patients with bipolar disorder or depression (36–38). No studies have explored the relationship between these three variables in PLWH. This study, which excluded participants diagnosed with depression or bipolar disorder, showed that most PLWH with depressive symptoms reported mild to moderate depressive symptoms. Our finding extends the application of social zeitgeber theory in the HIV context and confirms the direct effect of depressive symptoms on sleep in PLWH. Although this study shows that the severity of depressive symptoms predicts sleep quality, the opposite direction of causality is equally plausible, suggesting that future longitudinal studies are needed to explore the causality and directionality between variables in PLWH. Regardless of causality among variables, we also found a high co-occurrence of poor sleep and depression in PLWH, suggesting that both should be assessed and effectively treated to prevent or mitigate the effects of the other.

We found that social support and coping styles directly affect social rhythms and depression and indirectly affect sleep quality through social rhythms and depression. Hypotheses 2 and 3 were confirmed. Previous research revealed that high social support was correlated with low social rhythm regularity (80) and that problem-focused active coping was associated with more physical activities (81). When individuals face external stressful events, effective social support and coping styles make the social zeitgeber less likely to be disrupted, and individuals tend to maintain their original daily activities (80, 81). Sexual and HIV-related stigma and discrimination remain a source of social vulnerability and social isolation for PLWH, which may lead to reduced perceived social support and greater vulnerability to passive coping styles (82, 83), resulting in a series of problems such as depression or sleep disturbances (84–86). The pathway model in this study suggests that social rhythm is a potential intervening variable and that simple lifestyle changes may improve depression and sleep in PLWH. However, social support and coping styles explained only 4.4% of the variance in social rhythms within the model. Antiretroviral therapy is a social zeitgeber specific to PLWH, and PLWH need to take medication regularly every day (17), which may also be a key antecedent affecting the stability of social rhythms. During the COVID-19 pandemic, social restriction policies were implemented to minimize COVID-19 transmission. The regulations may weaken the social zeitgeber information via decreased stability of the timing of daily activities in PLWH, such as sleeping, school/work, socializing, eating, entertainment, and physical activity, which further lead to mental health disorders, such as anxiety and depression (22, 87). Future research needs to further explore specific factors affecting social rhythm in PLWH, such as antiretroviral therapy, stigma, interpersonal interactions, and the COVID-19 pandemic.

Several limitations should be noted. First, although we identified the optimal path for the unidirectional effect of sleep quality on depression using an automated model specification search function, the cross-sectional design precludes making assumptions about causality among these factors. There may be bidirectional relationships between social rhythms, depression, and sleep, and longitudinal studies are needed to explore the causality and directionality among variables. Second, to explore the applicability of the social zeitgeber theory in PLWH, we only explored the theory-based psychosocial factors in the path model. However, factors such as age, the length of time since HIV diagnosis, and infection route may also be important factors that affect the sleep of PLWH. Future studies should consider other confounders that may affect sleep in PLWH beyond the theory. Third, this study only recruited participants from a designated hospital for HIV/AIDS diagnosis and treatment and the Center for Disease Control and Prevention in Changsha City, Hunan Province. Given the differences in medical and economic levels between regions, the representativeness of the sample and the generalization of the results may be limited to a certain extent. In the future, multi-center research should be carried out to verify the relationship between variables. Finally, this study used only self-reported subjective tools to measure sleep and social rhythms, which may have recall bias (88). Future research using objective measurement tools such as actigraphy can make up for the shortcomings of existing subjective measures.

Despite the aforementioned limitations, this study investigates the influencing factors of sleep in PLWH based on social zeitgeber theory. Our study reveals that social rhythms, sleep, and depression is theoretically linked in a complex way. Although the direction of causality remains to be tested, the association between social rhythms, sleep quality, and depression in the pathway model of our study provides new insights into future intervention development, suggesting that interventions for stabilizing social rhythms show the potential to improve sleep and depression in PLWH. Future studies need to explore in depth the role and specific influencing factors of social rhythms in PLWH, and interventions such as interpersonal and social rhythm therapy targeted at improving interpersonal communication and stabilizing social rhythms may bring breakthroughs in the intervention development of sleep disturbances in PLWH.

## Conclusion

This study extends the applicability of the social zeitgeber theory in the HIV context. Social rhythms, sleep, and depression is not simply linked in a cascading sequence but is theoretically linked in a complex way. Our results show that social rhythms have direct and indirect effects on sleep in PLWH, and the relationship suggests that lower social rhythm stability is associated with poorer sleep quality. Although longitudinal studies are needed to test the causality and directionality of these associations, our results suggest that intervention strategies aimed at stabilizing social rhythms have the potential to improve sleep. More studies are needed to explore the role and influencing factors of social rhythms and to develop interventions to stabilize the daily activities of PLWH.

## Data availability statement

The datasets presented in this article are not readily available because to maintain the privacy of people living with HIV, data is presented in a total sample format. Requests to access the datasets should be directed to JM, 295679634@qq.com.

## Ethics statement

The studies involving human participants were reviewed and approved by the Institutional Review Board of Central South University. The patients/participants provided their written informed consent to participate in this study.

## Author contributions

JM: conceptualization, data curation, formal analysis, funding acquisition, and writing–original draft. XX: investigation, methodology, and writing–review and editing. WW: methodology, supervision, and writing–review and editing. YiJ and YaJ: writing–review and editing. HW: conceptualization, supervision, funding acquisition, and writing–review and editing.

## Funding

This work was supported by the Provincial Natural Science Foundation of Hunan Grant (no. 2022JJ30769); the Hunan Provincial Innovation Foundation for Postgraduate (no. CX20210113); and the Innovation-driven project of Central South University (no. 2021zzts0334). The funding sources had no role in the study design; the collection, analysis, and interpretation of data; the writing of the review; or the decision to submit the paper for publication.

## Conflict of interest

The authors declare that the research was conducted in the absence of any commercial or financial relationships that could be construed as a potential conflict of interest.

## Publisher’s note

All claims expressed in this article are solely those of the authors and do not necessarily represent those of their affiliated organizations, or those of the publisher, the editors and the reviewers. Any product that may be evaluated in this article, or claim that may be made by its manufacturer, is not guaranteed or endorsed by the publisher.
